# Lifestyle modification awareness and its association with biochemical control among diabetes mellitus patients: A mixed-methods study

**DOI:** 10.6026/973206300222000

**Published:** 2026-04-30

**Authors:** Prasun Saluja, Pawan Kumar, Shipa Deoke, Jaya Sai Nadella, Dhivya Mohan

**Affiliations:** 1 Department of Medicine, Saraswathi Institute of Medical Sciences, Hapur, New Delhi, India; 2 Department of Acute Medicine, Junior Specialist Doctor, Heartlands Hospital Birmingham UK, West Midlands, United Kingdom; 3 Department of Medicine, NKP Salve Institute of Medical Sciences & RC and Lata Mangeshkar Hospital, Nagpur, India; 4 Department of General Medicine, Jersey General Hospital, Jersey Channel Islands, The Parade, St Helier, Jersey JE1 3QS, Jersey; 5 Department of Medicine, Aarupadai Veedu Medical College and Hospital, Pondicherry, India

**Keywords:** Diabetes mellitus, lifestyle change, knowledge, mixed-methods study, biochemical measurements, glycemic control, patient education, health behaviour, self-management, public health

## Abstract

Poor adherence to lifestyle modification remains a major barrier to optimal glycemic control in diabetes mellitus. Therefore, it is of interest to assess lifestyle awareness and behaviours among 200 adults with diabetes and examined their association with biochemical parameters. Although 79% recognized the importance of diet and 67% acknowledged physical activity, only 28% reported daily exercise. Elevated fasting glucose and HbA1c were observed in 74% and 78% of participants, respectively and awareness scores showed significant negative correlations with HbA1c (r = -0.38, p < 0.001). Thus, we show a persistent gap between awareness and behavioural implementation and support structured, sustained lifestyle counselling in diabetes management.

## Background:

Diabetes mellitus is a chronic metabolic disorder characterized by persistent hyperglycemia due to impaired insulin secretion, insulin action or both [1]. Its global prevalence continues to rise, driven by urbanization, sedentary behaviour and dietary transition [2]. Poor glycemic control increases the risk of cardiovascular disease, nephropathy, neuropathy and retinopathy [3]. Lifestyle modification remains a cornerstone of diabetes management [4]. Dietary regulation, regular physical activity, weight reduction and stress control improve insulin sensitivity and metabolic outcomes [5]. Clinical guidelines consistently emphasize behavioural intervention alongside pharmacotherapy [6]. However, adherence to recommended lifestyle measures remains suboptimal in many populations [7]. Awareness of lifestyle recommendations does not always translate into sustained behavioural change [8]. Cultural dietary practices, limited health literacy, motivational decline and long disease duration may reduce adherence [9]. Inadequate self-monitoring and inconsistent follow-up further compromise metabolic control [10]. Biochemical parameters such as fasting glucose, HbA1c and lipid profile provide objective measures of adherence and disease management [11]. Mixed-method approaches allow simultaneous assessment of quantitative behavioural patterns and qualitative determinants of adherence. Correlating awareness scores with biochemical outcomes enables evaluation of the practical impact of lifestyle knowledge. Region-specific data are essential for designing targeted educational strategies [12]. Therefore, it is of interest to evaluate lifestyle modification awareness in individuals with diabetes mellitus and examine its association with biochemical parameters of metabolic control.

## Materials and Methods:

A cross-sectional mixed-method study was conducted among adults with diabetes mellitus attending selected outpatient clinics during the defined study period. Participants aged 18 years or older with a confirmed diagnosis of diabetes for at least six months were recruited consecutively during routine visits. Individuals with acute complications or cognitive impairment were excluded. Written informed consent was obtained prior to enrolment. Quantitative data were collected using a structured and validated questionnaire assessing awareness, dietary practices, physical activity, medication adherence and self-care behaviours. The tool was reviewed by subject experts and pilot tested before implementation. Sociodemographic and clinical characteristics were also recorded. Fasting blood glucose, HbA1c and lipid profile values were obtained from recent laboratory reports or measured on-site following standardized institutional protocols. A purposively selected subgroup participated in semi-structured interviews to explore barriers and facilitators related to lifestyle modification. Interviews were conducted by trained investigators and analysed using thematic analysis. Quantitative data were analysed using descriptive statistics and correlation analysis to examine associations between awareness scores and biochemical parameters. Statistical significance was set at p < 0.05. Ethical approval was obtained from the Institutional Ethics Committee and confidentiality was maintained throughout the study.

## Results:

The study included 200 individuals with diabetes mellitus. Most participants were between 41-60 years of age and males constituted 59% of the cohort. Nearly half had diabetes for 5-10 years and 62% were managed with oral hypoglycemic agents alone. Overall awareness of lifestyle modification was moderate; however, behavioural adherence was substantially lower across dietary, physical activity and self-care domains. Biochemical evaluation showed poor glycemic control in a majority of participants, with 74% exhibiting elevated fasting glucose and 78% having deranged HbA1c levels. Significant negative correlations were observed between awareness scores and key biochemical parameters. Participants with longer disease duration demonstrated lower adherence levels. Qualitative findings identified knowledge gaps, motivational barriers and socio-cultural influences as major determinants of lifestyle behaviour. Table 1 (see PDF) shows that the majority of participants were aged 41-60 years (69%); males constituted 59% and 40% had graduate-level education. Table 2 (see PDF) indicates that 44% had diabetes for 5-10 years, 62% were managed with oral hypoglycaemic agents alone and 73% reported a family history of diabetes. Table 3 (see PDF) demonstrates that awareness of dietary modification was highest at 79%, followed by physical activity at 67%, while awareness of stress management was lowest at 51%. Table 4 (see PDF) highlights that 46% consistently avoided sugary foods, 42% reported balanced meals and only 34% practiced portion control regularly. Table 5 (see PDF) compares physical activity patterns and shows that 28% engaged in daily exercise, while 37% exercised less than 20 minutes per session. Table 6 (see PDF) depicts good medication adherence in 61% of participants, whereas regular glucose monitoring was reported by 49% and adequate foot care by 42%. Table 7 (see PDF) shows that 74% had elevated fasting blood glucose, 78% had abnormal HbA1c levels, 44% had elevated total cholesterol and 57% had raised triglycerides. Table 8 (see PDF) indicates significant negative correlations between awareness score and fasting blood glucose (r = -0.32, p = 0.001), HbA1c (r = -0.38, p < 0.001) and triglycerides (r = -0.21, p = 0.012). Table 9 (see PDF) demonstrates a decline in good adherence from 47.2% among participants with diabetes duration <5 years to 35% among those with duration >10 years. Table 10 (see PDF) highlights qualitative themes including limited knowledge, motivational constraints, socio-cultural dietary influences and the positive impact of physician advice and family support.

## Discussion:

This mixed-method study evaluated awareness and adherence to lifestyle modification among individuals with diabetes mellitus and examined its association with biochemical outcomes. The findings demonstrate moderate awareness but inadequate behavioural implementation. A substantial proportion of participants recognized the importance of diet and physical activity. However, consistent adherence to recommended practices were limited [13]. The discrepancy between awareness and behaviour is clinically significant. Although 79% acknowledged dietary importance, only 46% consistently avoided sugary foods and 34% practiced portion control. Similarly, while 67% recognized the role of physical activity, only 28% reported daily exercise [14]. These findings indicate that knowledge alone does not ensure sustained behavioural change. Barriers such as motivation, time constraints and cultural dietary practices likely contribute to this gap. Biochemical findings reinforce this observation. Elevated fasting glucose and HbA1c were observed in 74% and 78% of participants, respectively. These values indicate suboptimal glycemic control despite ongoing treatment. Lipid abnormalities were also prevalent. The high burden of metabolic derangement reflects inadequate integration of lifestyle modification into daily practice [15]. The significant negative correlations between awareness scores and HbA1c, fasting glucose and triglycerides are noteworthy. Participants with higher awareness demonstrated better metabolic profiles. Although correlation does not imply causation, the association suggests that improved understanding may support better self- management. Awareness appears to influence behavioural patterns that translate into measurable biochemical outcomes [16]. Disease duration influenced adherence patterns. Participants with diabetes for more than 10 years showed lower rates of good adherence. This may reflect behavioural fatigue, therapeutic complexity or declining motivation over time. Chronic disease management requires sustained reinforcement rather than episodic counselling. Periodic education and structured follow-up may mitigate long-term decline in adherence [17]. Self-care practices were inconsistent. Medication adherence was relatively higher compared to glucose monitoring and foot care. This pattern suggests that pharmacological adherence is prioritized over lifestyle and preventive measures. However, comprehensive diabetes management requires integration of medication, monitoring and behavioural strategies [18]. Qualitative findings provided contextual depth. Participants reported limited clarity regarding portion sizes and culturally appropriate dietary substitutions. Time constraints and occupational demands limited physical activity. Social events and family food patterns influenced dietary compliance. Facilitators included physician guidance, peer support and visible health improvement. These findings emphasize that behavioural change is influenced by social and environmental determinants [19]. The mixed-method design strengthens interpretation. Quantitative analysis identified measurable gaps in awareness and biochemical control. Qualitative insights explained underlying behavioural barriers. Correlating awareness scores with objective metabolic markers enhances the clinical relevance of the findings [20]. This study contributes region-specific evidence linking lifestyle awareness to biochemical outcomes in diabetes. Few studies have integrated behavioural assessment with objective metabolic parameters using a mixed-method framework. The findings highlight that moderate awareness is insufficient to achieve optimal metabolic control. Structured behavioural counselling and culturally tailored interventions are required to bridge the knowledge-practice gap. Limitations include cross-sectional design and reliance on self-reported behavioural data. Causal relationships cannot be established. Nevertheless, objective biochemical assessment strengthens the validity of observed associations. Overall, diabetes management must extend beyond pharmacotherapy. Sustained behavioural reinforcement, culturally sensitive dietary counselling and long-term follow-up are essential. Enhancing awareness is necessary but must be accompanied by structured strategies that support consistent lifestyle implementation.

## Conclusion:

Lifestyle modification awareness among individuals with diabetes mellitus is moderate, but sustained behavioural adherence remains inadequate. Higher awareness levels are associated with better biochemical control, yet a significant knowledge-practice gap persists. Long-standing disease duration further reduces adherence to recommended lifestyle behaviours. Thus, structured, culturally tailored and periodically reinforced behavioural interventions are necessary to improve long-term metabolic outcomes.
